# Dimensional Deviations of Horizontal Thin Wall of Titanium Alloy Ti6Al4V Determined by Optical and Contact Methods

**DOI:** 10.3390/ma16237272

**Published:** 2023-11-22

**Authors:** Szymon Kurpiel, Krzysztof Zagórski, Jacek Cieślik, Krzysztof Skrzypkowski, Sarken Kapayeva, Maral Torekhanova

**Affiliations:** 1 Faculty of Mechanical Engineering and Robotics, AGH University of Science and Technology, Mickiewicza 30 Av., 30-059 Krakow, Poland; 2 Faculty of Civil Engineering and Resource Management, AGH University of Science and Technology, Mickiewicza 30 Av., 30-059 Krakow, Poland; skrzypko@agh.edu.pl; 3 International School of Engineering, East Kazakhstan Technical University, Ulitsa Serikbayeva 19, Ust-Kamenogorsk 070000, Kazakhstan; sarkenkapayeva@yahoo.com (S.K.);

**Keywords:** horizontal thin-walled sample, milling, titanium alloy, Ti64Al4V, adaptive milling, deviation, optical method, contact method, 3D optical scanner, coordinate measuring machine

## Abstract

Thin-walled structures are used in many industries. The need to use such elements is dictated by the desire to reduce the weight of the finished product, as well as to reduce its cost. The most common method of machining such elements is the use of milling, which makes it possible to make a product of almost any shape. However, several undesirable phenomena occur during the milling of thin-walled structures. The main phenomenon is a deformation of the thin wall resulting from its reduced stiffness. Therefore, it is necessary to control the dimensional and shape accuracy of finished products, which is carried out using various measuring instruments. The development of newer measuring methods such as optical methods is being observed. One of the newer measuring machines is the 3D optical scanner. In the present experiment, thin-walled samples in horizontal orientation of Ti6Al4V titanium alloy were machined under controlled cutting conditions. During machining, the cutting speed and feed rate were assumed constant, while the input factors were the tool and cutting strategy. This paper presents graphs of deviations in the determined cross-section planes of thin-walled structures using a 3D optical scanner and a coordinate measuring machine. A correlation was made between the results obtained from the measurement by the optical method and those determined by the contact method. A maximum discrepancy of about 8% was observed between the methods used.

## 1. Introduction

The driving factors behind the use of thin-walled components in many industries are the desire to reduce the weight of structures and lower costs. The realization of these trends is implemented, e.g., by reducing machining time, which forces the use of increasingly newer machining methods or higher values of machining parameters. Available tools, geometrically designed for different strategies, allow working with similar and increased values of cutting parameters [1,2]. Consequently, cutting conditions that allow machining with increased cutting speeds or feed rates are being sought. Despite the advanced level of machining, including thin-walled structures, there are still many limitations that are being analyzed by researchers. One of the main problems of machining thin-walled products is the elastic deformation of the wall during machining, which leads to deformation, resulting in geometric errors and vibrations and deterioration of the quality of the workpiece [3,4,5,6,7,8]. This is due to the low stiffness of the workpiece through the small cross-sectional area of the thin wall. Shape deformation is a serious problem as aerostructure manufacturers strive to improve the quality of their products to remain competitive. This involves the use of increasingly narrower dimensional tolerances [9,10,11]. Based on this, it is concluded that it is necessary to conduct research in this area to determine the effect of cutting conditions on the dimensional accuracy of finished products. In the aspect of the deformation of thin-walled structures, the key is the study of dimensional accuracy, which is the process of measuring and evaluating the conformity of the actual object and surface shape from the assumed model. The results from such measurements are used to assess the quality of manufactured products, and compliance with the assumed requirements and can be the basis for determining the causes of nonconformity, as well as control of production processes. These tests have become crucial in many manufacturing processes due to the stringent requirements for objects. It is almost impossible to obtain a product with the theoretically assumed dimensions, so differences appear between the assumed and obtained shape and dimensions of the finished product [12]. These differences are referred to in terms of deviations, which are positive sign (material allowance) or negative sign (material loss) values of manufacturing error. Geometric accuracy is characterized by errors in dimension, position, and shape, which are factors that affect the functioning of machine components. Occurring errors in the manufacturing process affect the formation of uneven clearances and indentations in joints and uneven stress distribution and ultimately reduce the service life of machine components [13]. 

Dimensional and shape accuracy tests are carried out using either the non-contact or the contact method. Within these methods, various machines are available to check deviations. Of particular importance in today’s industry are coordinate measuring machines (CMMs), which check specific features of the finished product in an automated method. 

The main type of non-contact measurement is the use of optical methods, for which there is continuous development in the field of metrology. Optical methods are noted for their high efficiency and the ability to measure components with complex shapes. One of the strongly developing optical methods is 3D optical scanner measurement. Mechanical contact measurement systems collect data in a linear or point-like manner, while optical measurement systems provide deviations between actual coordinates and model values. Measuring data obtained with an optical scanner contains all the information about the object, so the software retrieves detailed information about, e.g., GD&T, trimming, and hole positions. The Global Optical Measurement (GOM) method is a modern technology that allows precise measurements of product geometry and uses advanced cameras and image analysis software to record and analyze results related to object shape [2,14].

Within contact measurement, the most popular test machine is the coordinate measuring machine (CMM), which is one of the most accurate used in current metrology. The reason for its popularity is that it provides the ability to perform measurements with high precision for workpieces of virtually any shape [15]. A common feature of coordinate measuring machines is point-by-point measurement according to the established measurement program. At the very beginning, a product is made according to the assumed design, and then the measurement strategy is defined, during which the points to be measured are determined. In a further step, the coordinates of the points in space are measured, and the mutual orientation of the associated elements is determined and compared with the assumed model features. The result of these procedures is the representation of deviations, which are used to check whether the geometry of the fabricated object is aligned with the assumptions [16,17,18].

The results of the dimensional inspection of thin-walled structures in different orientations are observed in scientific publications that use different methods and measuring instruments. 

The authors of [19] analyzed the deviations of vertical thin-walled parts made from EN AW7075-T651 aluminum alloy using conventional milling and high-speed milling for finishing. The result of their research was a measurement of deviations made using a coordinate measuring machine. From the data presented, it was observed that the cutting method has a significant effect on the deviation values obtained. Another important conclusion from this work is the variable value of deviations on the cross-section of the sample. This shows that taking measurements in many areas is reasonable when evaluating surface quality.

Gang, in his work [20], presented the deflection of thin-walled titanium alloy parts in a vertical orientation for only one side of the machining surface. He showed a maximum deflection of the part of about 0.1 mm in the center of the wall, whose thickness after milling was equal to 2.5 mm.

In the study [21], thin-walled samples from titanium alloy Ti6Al4V were fabricated using various support methods. In the experiment, cylindrical face milling was used during one-sided machining of the thin-wall sample. The unsupported thin-wall machined sample was made with the following cutting parameters: V_c_ = 40 m/min, a_p_ = 16 mm, a_e_ = 0.2 mm, f_z_ = 0.06 mm/rev for a tool with a diameter of D = 12 mm. Based on the measurement, it is noticeable that the thin wall deviated by a value of about 0.1 mm at a maximum height of 30 mm.

Yusop et al. [22] presented cylindrically curved vertical thin-walled titanium alloy Ti6Al4V samples made using a trochoidal milling strategy. The shape of the samples used increased the rigidity of the machined surface. During machining, a milling tool with a diameter of 10 mm was used and the cutting speed V_c_ was adopted in the range of 47÷50 m/min (depending on the case), with the feed per tooth equal to f_z_ = 0.03 mm/rev. A larger section of the cut layer was used, as the overlap for trochoidal milling was 1.6 mm, while the depth of cut was 5 mm. The result of their study was a representation of the dimensional deviation at the mid-height of the sample on both sides, with the maximum deviation observed being +0.18 mm.

In the work [23], a radial depth of 0.2 mm and a cutting depth of 20 mm were used in the milling of a vertical thin wall of Ti6Al4V titanium alloy. The machining was carried out using a four-blade milling tool with a diameter of 12 mm at different configurations of cutting speed (90 m/min and 120 m/min) and feed rate (0.25 mm/rev and 0.35 mm/rev). The results of these tests were the roughness Ra (values in the range of 2.445 ÷ 3.109 μm were obtained) and dimensional deviations in vertical planes to the bottom of the sample (values in the range of about 0.06 ÷ 0.34 mm were obtained).

Zha et al. [24] presented the results of a study containing dimensional deviations of fan blades made of titanium alloy Ti6Al4V with a thin-wall thickness of 3.8 mm. A maximum deformation of the finished product of +0.21 mm was obtained. A ball milling cutter with a ball radius of R6 was used for finishing, and the following cutting conditions were adopted: V_c_ = 75 m/min, f_z_ = 0.0375 mm/rev, a_e_ = 0.01 mm. The cutting depth a_p_ values were varied according to the case and were 20 mm, 40 mm, 60 mm, and 75 mm. The measurement of deviations was carried out using a 3D optical scanner from Atos, which presents the possibility of using this instrument to measure thin-walled structures with larger dimensions; the tested object had dimensions of 210 × 210 × 200 mm.

The main goal of this work is to evaluate the dimensional deviations of titanium alloy samples containing a thin wall in horizontal orientation, milled under controlled cutting conditions. In the above publications, deviations are presented mainly for thin-wall structures made of aluminum alloy; fewer studies present the control of dimensional deviations for thin-walled titanium alloy structures. Usually, structures were studied in vertical orientation, while the established work focused on studying thin-walled structures in horizontal orientation.

An additional objective is to evaluate the feasibility of using a 3D optical scanner to measure thin-walled components of small dimensions. In the available studies, it is observed that the use of the 3D optical scanner for assessing the deformation of thin-walled structures is not common in scientific research [3,24]. A small number of studies are available that present the use of this instrument during the inspection of dimensional and shape accuracy. Measurement of thin-walled structures using this method is difficult to perform, and the available studies do not present a correlation with commonly used measurement methods such as contact CMM measurements. Confirmation of the possibility of measurement by such a method (3D optical scanner GOM) with sufficient measurement accuracy will be valuable information. Available studies that include measurements with a 3D optical scanner focus on thin-walled products with larger dimensions. Therefore, it was decided to cross-correlate the results obtained from the study of the dimensional and shape accuracy of samples obtained by the non-contact (optical) method using a 3D optical scanner (GOM) with those obtained by the contact method using a Zeiss coordinate measuring machine. In the paper [2], we addressed the problem of measuring the deviations of thin-walled structures in vertical orientation with small dimensions using a 3D optical scanner. Within the framework of this study, we were able to determine the dimensional deviations, but we did not present the correlation of the results obtained from the measurement by the optical method with other popular methods.

## 2. Materials and Methods

### 2.1. Experimental Setup and Milling Conditions

A series of samples with a thin wall in horizontal orientation were machined on a Mikron VCE 600 Pro machining center manufactured by GF Machining Solutions (Switzerland), which was equipped with iTNC 530 control software produced by Heidenhain (Germany). 

**Figure 1:**
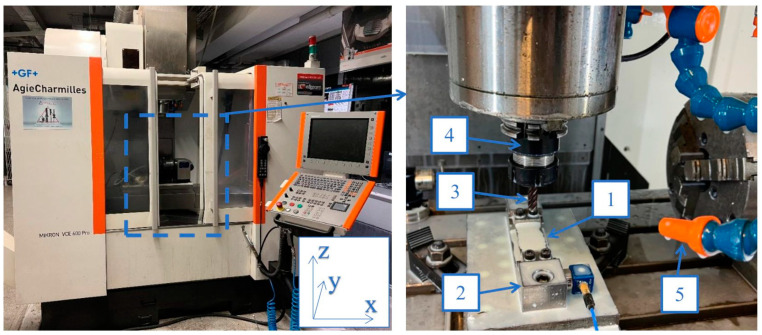
Experimental setup: 1—sample; 2—adaptor; 3—tool; 4—tool holder; 5—coolant.

During the machining of all the samples, an identical method of mounting monolithic milling cutters in a ∅10-precision sleeve and placing them in an ER32 tool holder was used (Figure 1). Milling was carried out using SILUB MAX water–oil emulsion, which is a two-component coolant that meets the requirements of TRGS 611 according to the recommendations of the tool manufacturer. During the experiment, a mixture of 15% oil emulsion and 85% water was used, as recommended by the coolant manufacturer. The coolant is designed for universal applications, including machining special materials under extreme conditions [25]. Cubes for machining workpieces with thin horizontal walls were mounted to a pre-prepared adaptor, which was bolted to the dynamometer using two M10 screws (Figure 1).

**Figure 2:**
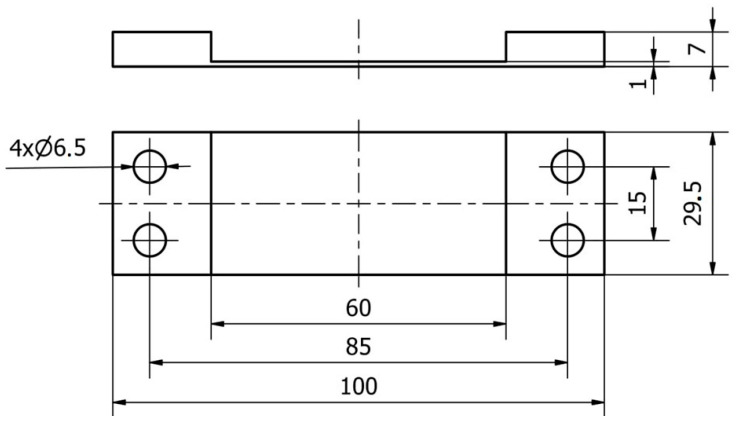
Documentation of the horizontal thin-walled sample.

A semi-finished product with dimensions of 7 × 29.5 × 100 mm was used to prepare the samples by milling treatment. The finished product has a thin wall with a thickness of 1 mm over a length of 29.5 mm and a width of 60 mm (Figure 2).

The machining of samples was carried out under controlled cutting conditions, for which the input factors were cutting tools and cutting strategy. A description of the tools and the cutting strategy used for each sample is presented in Table 1.

Within the first input factor, three tools were used, geometrically designed for different machining methods: for general purposes (capable of machining all material groups), JS554100E2R050.0Z4-SIRA; for high-performance machining (designed for machining titanium alloys and nickel superalloys), JS754100E2C.0Z4A-HXT; for high-speed machining (designed for machining titanium alloys and nickel superalloys), JH730100D2R100.0Z7-HXT. 

The JS554100E2R050.0Z4-SIRA monolithic face milling cutter is a four-blade tool from the Jabro Solid^2^ family with geometry type JS554, containing one central blade. It is designed for general (universal) machining with increased material removal rates. The cutter has a two-stage core with variable pitch. The blades have a lead angle of 0° and a corner rounding of 0.5 mm, and the flange helix angle is 48°. Installation of the tool in the tool holder is carried out with a cylindrical shank, the diameter of which is 10 mm. Between the working part and the shank part, there is a 0° constriction with a diameter of 9.5 mm over a length of 29 mm. The working part is coated with a SIRON-A coating. An additional advantage of the tool is the chip splitter, which creates short chips during machining. It has a more positive geometry of the front teeth. The JS554 series milling cutters allow the machining of virtually all types of materials, including titanium alloys and nickel alloys [26].

The JS754100E2C.0Z4A-HXT monolithic face mill has the JS754 geometry type and is a tool from the Jabro Solid^2^ family. The tool has 4 cutting edges with a 0° lead angle. The blades have a 0.125 × 45° corner chamfer. The cutter has a flute helix angle of 48°. Between the working part and the shank, there is a constriction with a constriction angle of 0° and a diameter of 9.5 mm over a length of 29 mm. The working part is coated with HXT coating. The tool is mounted in the tool holder using a cylindrical shank with a diameter of 10 mm, and the shank itself is contained in tolerance class h5. The JS754 series cutters increase machining stability and tool life. They are characterized by a shorter working length, which increases strength and rigidity during machining and also ensures full engagement of the cutting edge. Using the aforementioned cutter, it is possible to machine specialty materials, including titanium alloys and nickel alloys [27].

The JH730100D2R100.0Z7-HXT monolithic face mill is a JH730 geometry tool from the Jabro Tornado family. The tool contains 7 cutting edges with unevenly spaced blades and no center blade. The blades, which have a lead angle of 0°, have a rounding at the corners equal to 1 mm. The flute helix angle for this tool is 34°. The cutter has no constriction between the working and shank parts and no internal cooling channel. It is characterized by a cylindrical shank with a diameter of 10 mm, for which the tolerance class is h5. The working part is covered with a polished HXT coating, and the cutter itself is designed for high-speed machining only of titanium alloys and nickel alloys. The tool is well suited for finishing external profiles [28].

**Figure 3:**
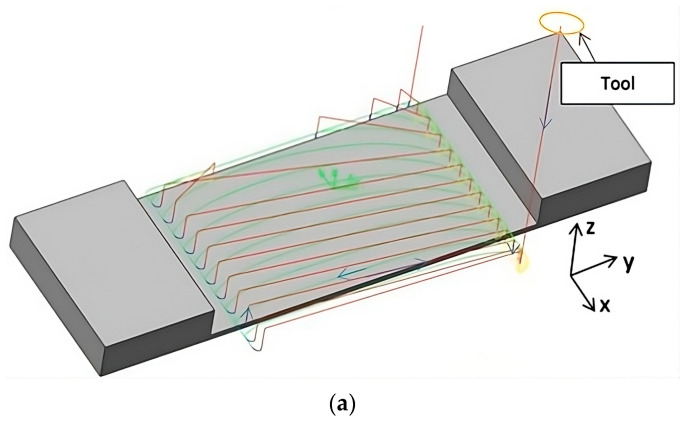

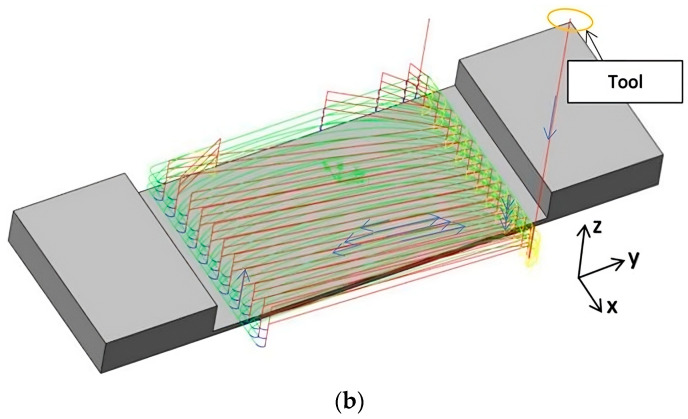
Tool paths during (**a**) adaptive face milling (increased radial depth) and (**b**) adaptive cylindrical milling (increased depth of cut)—the green color indicates the tool path during contact with the material, while the red color indicates the tool path during the return to the initial position.

The second input factor applied to machining thin-walled samples in horizontal orientation was the cutting strategy. Two approaches of adaptive milling were adopted: face milling (with increased involvement of the tool face) and cylindrical milling (with increased involvement of the cylindrical part of the tool). Adaptive milling is a relatively new machining method used for groove shaping, among other applications, to keep the tool in constant contact with the workpiece material for as long as possible. The strategy is derived from trochoidal milling, where the material is cut in an arc (maintaining a constant wrap angle) and returned in the shortest straight line. Table 1 shows the type of cutting strategy used for each sample, as well as the adopted cutting depth and radial depth values. The cutting paths of the described strategies are shown in Figure 3, where the green color indicates the tool path during contact with the material, while the red color indicates the tool path during the return to the initial position. Depending on the strategy adopted, different cutting depth and radial depth values were used. For samples machined with adaptive face milling, a larger radial depth a_e_ value of 4 mm and a depth of cut a_p_ of 2 mm were assumed. In the case of adaptive cylindrical milling, a larger cutting depth value a_p_ of 6 mm and a radial depth a_e_ of 1.33 mm were assumed.

Based on the cutting tool manufacturer’s catalog, machining conditions and cutting parameters were selected. According to the assumptions adopted about similar machining conditions for the same tools, a cutting speed value of V_c_ was used that was in the middle of the recommended range and equaled 100 m/min. The feed rate V_f_ during the experiment was equal to 255 mm/min.

For each of the designated cases, a criterion of constant material removal rate was adopted, which equaled MRR = 2.03 cm^3^/min. The typical form of this rate is presented in Equation (1) [29,30].

MRR = V_f_·a_p_·a_e_ [cm^3^/min] (1)

To machine horizontal thin-wall samples, the titanium alloy Ti6Al4V, which is popular in the aerospace industry, was used [1,2,31,32]; its basic mechanical properties and chemical composition are shown in Table 2 and Table 3. Even though aluminum alloys play a significant role in aircraft, titanium alloys are still used for loaded aircraft components. Ti6Al4V titanium alloy was selected for the experiment because of its significant use in the aerospace industry. This trend will be observable in the future, due to the lack of developed alternative materials providing adequate properties where required. The material is mainly used in turbine components, where there is an increase in the use of thin-walled structures.

These materials are characterized by remarkable mechanical properties at sufficiently low density [33]. In the design of the F-41 fighter jet, the largest titanium alloy component is its caisson. The use of the material for this element made it possible to reduce the weight by about 410 kg [34]. Titanium and nickel alloys have significant applications in structural components where resistance to high temperatures is required. These materials are most commonly found in engines and aircraft parts operating at elevated temperatures [35]. Titanium alloys can be found in turbine construction, with Ti6Al4V finding its way into both the aircraft structure itself and its engine. This alloy in its annealed state is suitable for applications where the temperature does not exceed 400 ℃ [36]. Titanium alloys belong to the group of difficult-to-machine materials due to their metallurgical properties, which require special machining techniques [37,38,39,40,41]. The main problem that occurs during milling is the tendency of the chip to seal the tool blades [32]. Additional factors that make machining difficult are low thermal conductivity and diffusivity, high stiffness, low elastic modulus, and high chemical reactivity at elevated temperatures [42].

**Table 2:** The mechanical properties of titanium alloy Ti6Al4V, grade 5 (own elaboration, based on [1,43]).

Mechanical Property	Density	Elongation at Break	Yield Strength 0.2%	Tensile Strength Rm
Value	4.52 g/cm^3^	min. 10%	min. 828 MPa	min. 895 MPa

**Table 3:** The chemical composition of titanium alloy Ti6Al4V, grade 5 (own elaboration, based on [1,43]).

Element	Ti	Al	V	Fe	O	C	N	H
Percentage (%)	other	5.5–6.75	3.5–4.5	≤0.4	≤0.2	≤0.08	≤0.05	≤0.015

**Figure 4:**
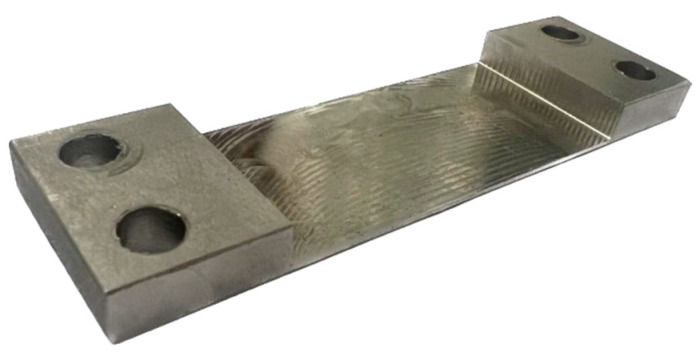
Representation of a sample with a thin wall in horizontal orientation.

Based on the established plan and the presented processing method and conditions, samples with a horizontal thin wall were made. The representation of the obtained sample is shown in Figure 4.

### 2.2. Benefits and Limitations of the Used Methods for Deviation Measurement

The obtained samples, the representation of which is shown in Figure 4, were subjected to measurement of dimensional and shape accuracy by two methods—using a 3D optical scanner and a coordinate measuring machine. Both methods have several advantages and benefits, but also disadvantages and limitations. A summary of such features and a mutual comparison of the measurement methods used are shown in Table 4.

### 2.3. Measurement of Deviations Using the 3D Optical Scanner

The measurement of dimensional and shape accuracy was carried out using the Atos ScanBox 6130 optical scanner, designed by GOM (Germany). The measurement program and series of measurements were prepared and carried out using the dedicated GOM Inspect 2020 (2020.0.4.135965) software.

**Figure 5:**
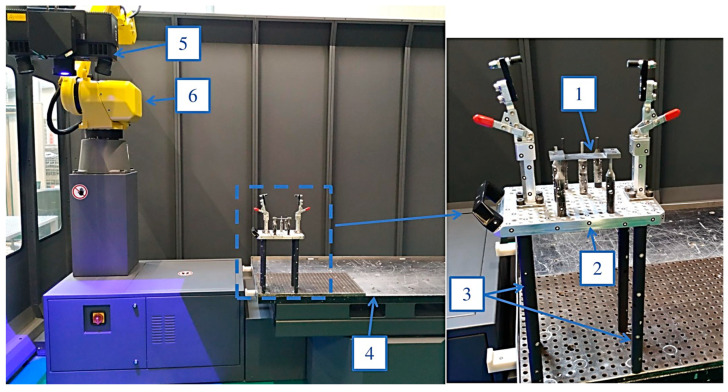
The scheme of measuring deviation using the 3D optical scanner: 1—thin-wall sample; 2—universal base; 3—machine posts; 4—rotary table; 5—GOM projector; 6—measuring arm.

The samples were mounted on a universal base, which was placed on the machine posts using magnets. The machine posts were bolted to the rotary table and were used to make it easier and more unobstructed for the measuring arm with the projector to access the measurement site, as well as to collect points in space from all sample locations. The mounting method was adopted based on the recommendations of the measuring machine manufacturer. The scheme with the description for measuring dimensional and shape accuracy using the 3D optical scanner is shown in Figure 5. 

**Figure 6:**
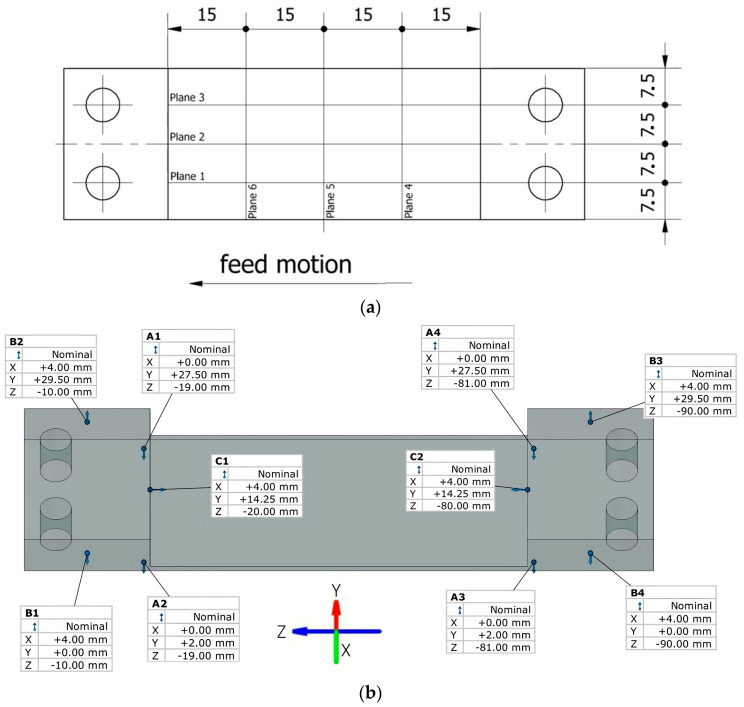
Description of measuring sections for samples with thin horizontal walls during GOM optical measurement: (**a**) location of measuring sections; (**b**) adopted method of basing the sample.

The measurement was carried out in the free state; none of the samples were immobilized. The sections for the sample were made in 6 planes perpendicular to the axis of the tool, determining the deflection arrow from both sides of the thin-walled surface—3 planes each in the directions parallel (planes 1–3) and perpendicular (planes 4–6) to the feed motion. From the machined side of the plane, the areas were numbered 1–6, while from the bottom of the sample, the areas were numbered 1′–6′. Figure 6 shows a graphical description of the planes along with the dimensions and adopted method of basing for the horizontal thin-walled sample during the optical method measurement.

Optical scanner measurement is a relatively new measurement method with some limitations and requires specific conditions. For successful measurement using this method, the tested surface has to be free of dirt and debris, as their presence can affect the accuracy of the results obtained. In the case of measuring products with small dimensions and/or thin walls, measurement becomes more challenging and problematic. In such cases, it is necessary to properly prepare the measurement program so that the intersections of the striations generated by the projector occur on the surface of the measured object, for which information on their distances and angles is obtained. Given the emerging limitations, it was decided to carry out the test by the contact method using a coordinate measuring machine, which is commonly used for dimensional and shape inspection of products. 

### 2.4. Measurement of Deviations Using a Coordinate Measuring Machine

Contact measurement of dimensional and shape accuracy using a Zeiss Contura (Germany) coordinate measuring machine (CMM) was carried out to check the correctness of the obtained deviations determined using the 3D optical scanner. The measurement program and reports were prepared in Zeiss Calypso 2020 software.

**Figure 7:**
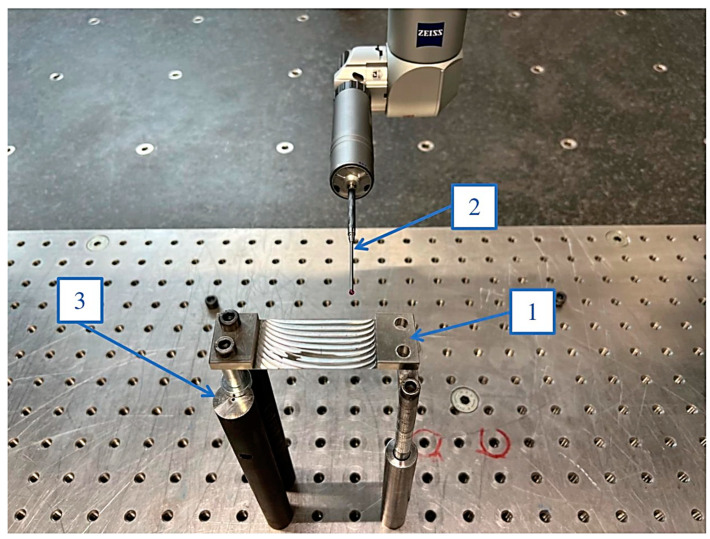
Measuring station for testing deviations by contact method using Zeiss Contura coordinate measuring machine: 1—sample; 2—measuring probe; 3—machine posts.

When measuring using the contact method with a coordinate measuring machine, the sample was placed on posts bolted to the CMM table to allow measurement of the bottom of the sample. The mounting method was comparable to the one adopted during measuring using the optical method. The measurement scheme for the contact method using a Zeiss Contura CMM is shown in Figure 7. Similarly to the measurement by the optical method, the test was carried out in the free state, and the screws appearing in Figure 7 were used only to secure the sample firmly to the machine posts.

**Figure 8:**
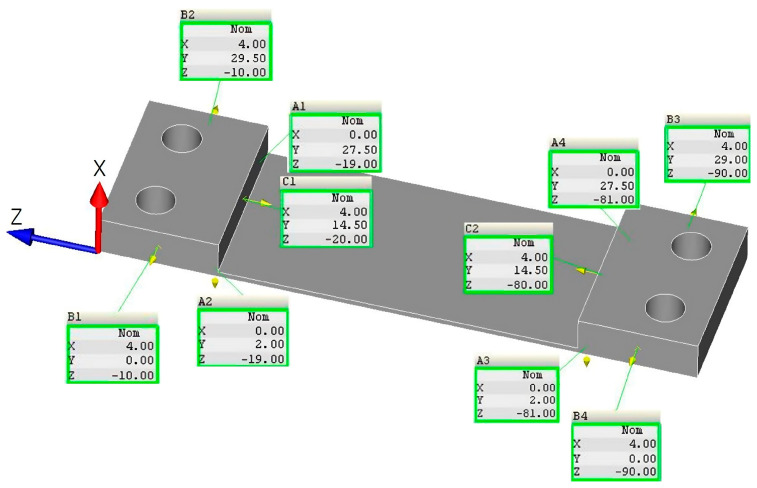
Adopted method of basing the sample during contact measurement using Zeiss Contura coordinate measuring machine.

The same measurement conditions were assumed during measuring with a 3D optical scanner; i.e., the same basing method was adopted (Figure 8) and the same measurement points were selected. The description of the measurement planes and their locations were adopted according to the data shown in Figure 6.

## 3. Results and Discussion

**Figure 9:**
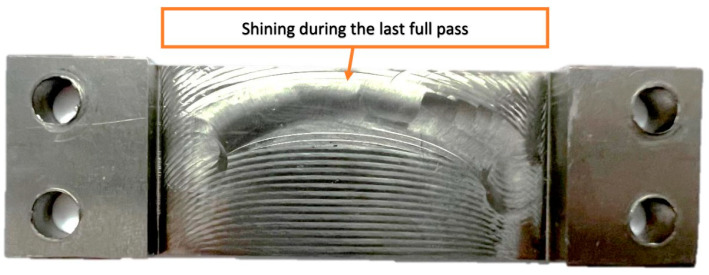
Shining of the machined surface of a sample with a horizontal thin wall appearing during the last full pass.

The manufactured samples with a horizontal thin wall presented a characteristic feature—a shining that appeared on the machined surface at the location of the last full pass (Figure 9). This feature was revealed for the samples milled using an adaptive cylindrical milling strategy. 

### 3.1. Results of Optical Method Measurement 

**Figure A1:**
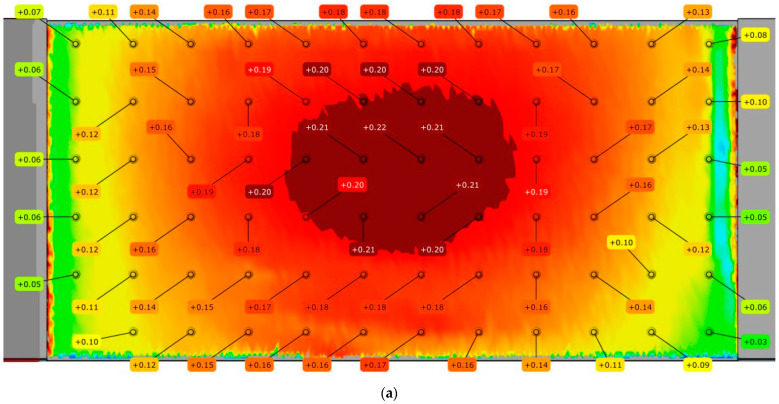

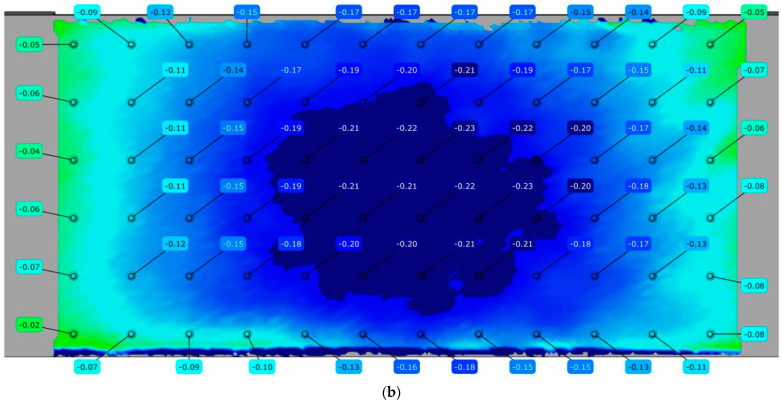
Example of GOM optical scanner measurement results for sample T1 with a horizontal thin wall: (**a**) color map—machined surface; (**b**) color map—bottom of the sample.

In the first part, the dimensional and shape accuracy of samples with thin walls in horizontal orientation was determined by the optical method using a 3D optical scanner (GOM), as described in Section 2.3. The most common result of measurements using a 3D optical scanner (GOM) is a color map, which represents the deviation of the finished product from the assumed shape. Examples of color maps obtained from optical scanner measurements for a sample with a horizontal thin wall are shown in the appendix in Figure A1. 

The results of the analyzed samples are presented graphically as a graph of the deviation of the thin wall depending on its length. The sign in front of the deviation value was consistently adopted as in the color maps in the measurement report. The sign placed only indicates the direction of the deviation. A negative sign indicates material loss, while a positive sign indicates material allowance.

**Figure 10:**
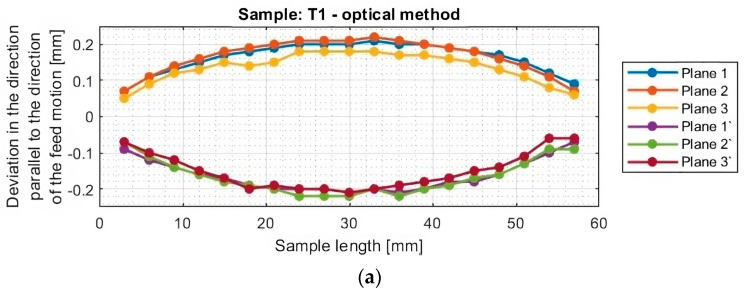

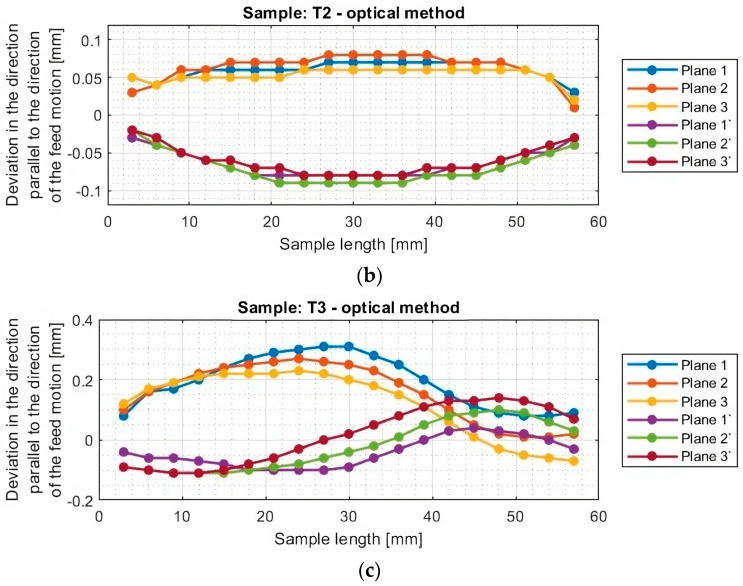
Deviations of the horizontal thin wall in the direction parallel to the direction of tool feed motion for titanium alloy samples machined using adaptive face milling strategy, determined by the optical method: (**a**) T1; (**b**) T2; (**c**) T3 (deviation graph for plane 2 taken from study [44]).

**Figure 11:**
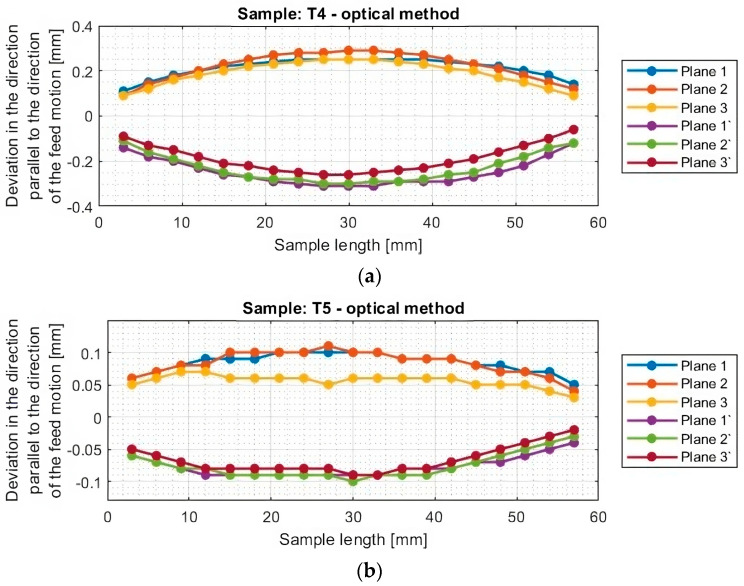

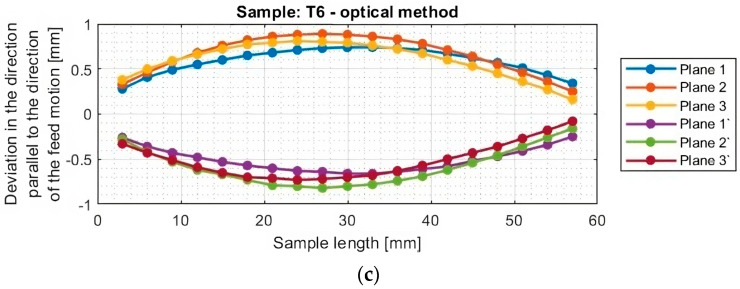
Deviations of the horizontal thin wall in the direction parallel to the direction of tool feed motion for titanium alloy samples machined using adaptive cylindrical milling strategy, determined by the optical method: (**a**) T4; (**b**) T5; (**c**) T6 (deviation graph for plane 2 taken from the study [44]).

Figure 10 and Figure 11 show the deviation plots of titanium alloy samples with horizontal thin walls in the direction parallel to the direction of the tool feed motion.

From the graphs for titanium alloy samples (T1–T6, excluding T3) shown in Figure 11, it can be observed that the values of deviations of the machined surface and those occurring from its bottom are almost symmetrical reflections concerning the horizontal axis y = 0. This symmetry is observed between planes occurring in the same section, i.e., between plane 1 and plane 1′, between plane 2 and plane 2′, and between plane 3 and plane 3′. This means that a thickness close to the assumed value of 1 mm was obtained. The exception is sample T3 machined with the tool for high-speed machining using adaptive face milling, for which the deviations are directed toward positive values. This means that a thickness of the thin wall in the horizontal orientation greater than assumed by about 0.2 mm was obtained.

From the graphs shown, it can be seen that the greatest deflection of each sample is seen in the middle of its length, that is, in the middle between the mounted points in the adapter. The nature and course of the graphs for samples T1, T2, and T4–T6 are similar among themselves. This is manifested in smooth graphs without significant anomalies. Sample T3 is characterized by a different graph course from the others; its machined surface is deformed in two directions. One of the reasons may be the effect of overheating of the sample; during the visual inspection, the occurrence of a characteristic scorching of the material surface was observed. Another factor affecting the obtained course of deviations may be the traces of material pulling out on the surface of the sample instead of shearing, which appear after crossing the center of the length of the sample. The reason for the aforementioned phenomena may be the clogging of the blades with the resulting chips or their improper discharge during machining with such a tool geometry.

**Figure 12:**
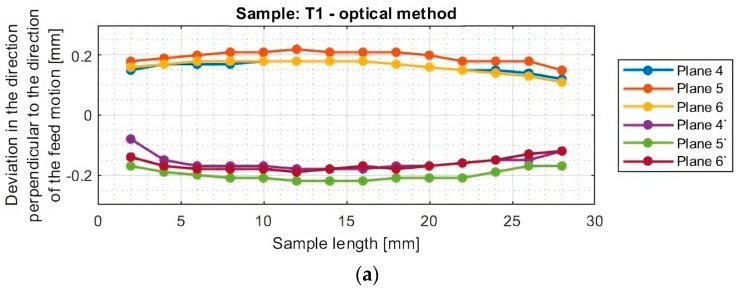

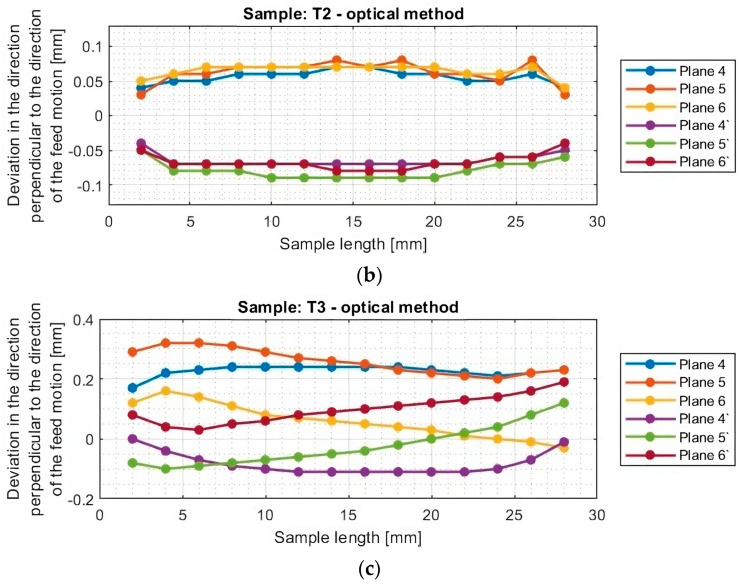
Deviations of the horizontal thin wall in the direction perpendicular to the direction of tool feed motion for titanium alloy samples machined using adaptive face milling strategy, determined by the optical method: (**a**) T1; (**b**) T2; (**c**) T3 (deviation graph for plane 5 taken from the study [44]).

In the next section, the deviations of titanium alloy samples with horizontal thin walls in the direction perpendicular to the direction of the tool feed motion were determined. A graphical representation of these results for each sample is shown in Figure 12.

**Figure 13:**
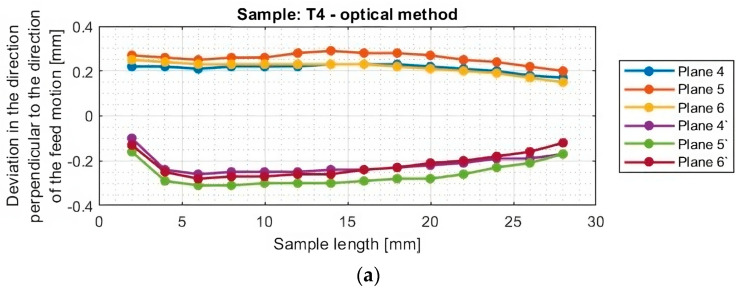

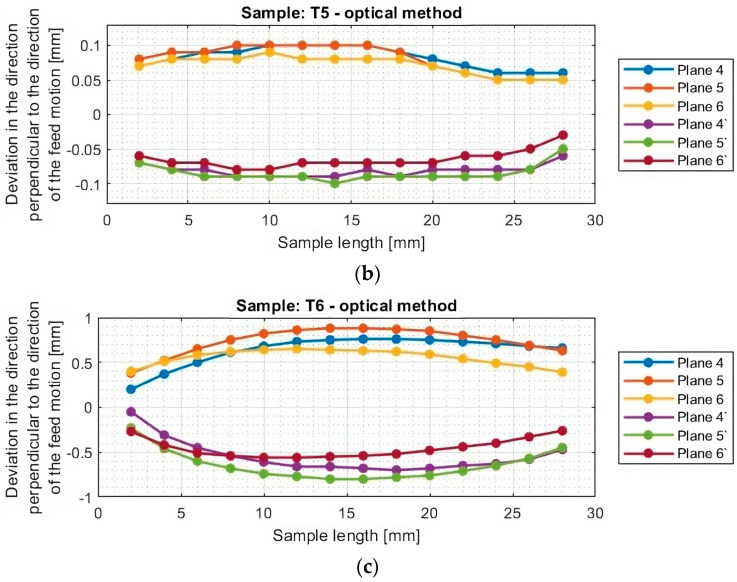
Deviations of the horizontal thin wall in the direction perpendicular to the direction of tool feed motion for titanium alloy samples machined using adaptive cylindrical milling strategy, determined by the optical method: (**a**) T4; (**b**) T5; (**c**) T6 (deviation graph for plane 5 taken from the study [44]).

The graphs of deviations in the perpendicular direction presented in Figure 13 confirm the relationship observed when analyzing the results in the parallel direction. One notices an approximately symmetrical distribution of deviations concerning the horizontal axis passing through the zero point (y = 0) for samples T1–T2 and T4–T6. The symmetrical distribution occurs between planes occurring in the same section, i.e., between plane 4 and plane 4′, between plane 5 and plane 5′, and between plane 6 and plane 6′. As previously mentioned, on this basis, it is concluded that the thickness of the thin wall is close to the assumed value of 1 mm. For sample T3, the shift of the graphs toward positive deviation values was confirmed, so a thin-wall thickness of about 0.2 mm more than assumed was obtained.

The graphs in Figure 12 and Figure 13 show that smaller deviations were obtained using the adaptive face milling strategy (T1–T3) compared to cylindrical milling (T4–T6). 

The use of the tool for general purposes during machining with the adaptive face milling (T1) presents sample deviations about 50% smaller compared to adaptive cylindrical milling (T4). The smallest deviations were obtained for samples machined with the tool for high-performance machining (T2 and T5), with smaller values of about 10% obtained for the adaptive face milling (T2) compared to cylindrical milling (T5). Milling with the tool for high-speed machining using adaptive face milling (T3) yields about 60% smaller results compared to adaptive cylindrical milling (T6). Sample T6 is characterized by much larger deviation values, reaching up to 1 mm, compared to the other samples. Although the graph is symmetrical concerning the horizontal axis passing through the zero point (y = 0), the difference in deviation values between the planes is up to 0.25 mm. 

Although the deviations for sample T3 do not reach the largest values, they are very unstable. The surface of this sample is severely deformed after machining, which also presents a very irregular graph shape that indicates that its thin-walled surface is twisted. Smooth graphs (without significant anomalies) and relatively stable values of surface deviations were observed for samples made with the tool for general purposes (T1 and T4) and the tool for high-performance machining (T2 and T5). The presented graphs for these samples, depending on their length, assume a near-horizontal shape, and their values in the range of the sample are comparable, without much mutual scatter between the planes.

The maximum deviation values of thin-walled samples in vertical orientation (of titanium alloy Ti6Al4V) in the cited publications are as follows: Zha [24] showed deviations equal to 0.21 mm, Yusop [22] showed deviations equal to +0.18 mm, Hintze [21] and Gang [20] obtained similar deviation values equal to 0.1 mm, and Polishetty [23] presented a maximum deviation equal to 0.34 mm (with a high roughness value—Ra_max_ = 3.109 μm). Comparing the cited deviation values, despite the use of a different orientation of the thin wall, with the maximum deviations obtained by the test performed, it can be seen that smaller values with a larger cross-section of the cut layer were obtained for sample T2 (max. deviation equal to 0.09 mm).

### 3.2. Results of Contact Method Measurement

**Figure 14:**
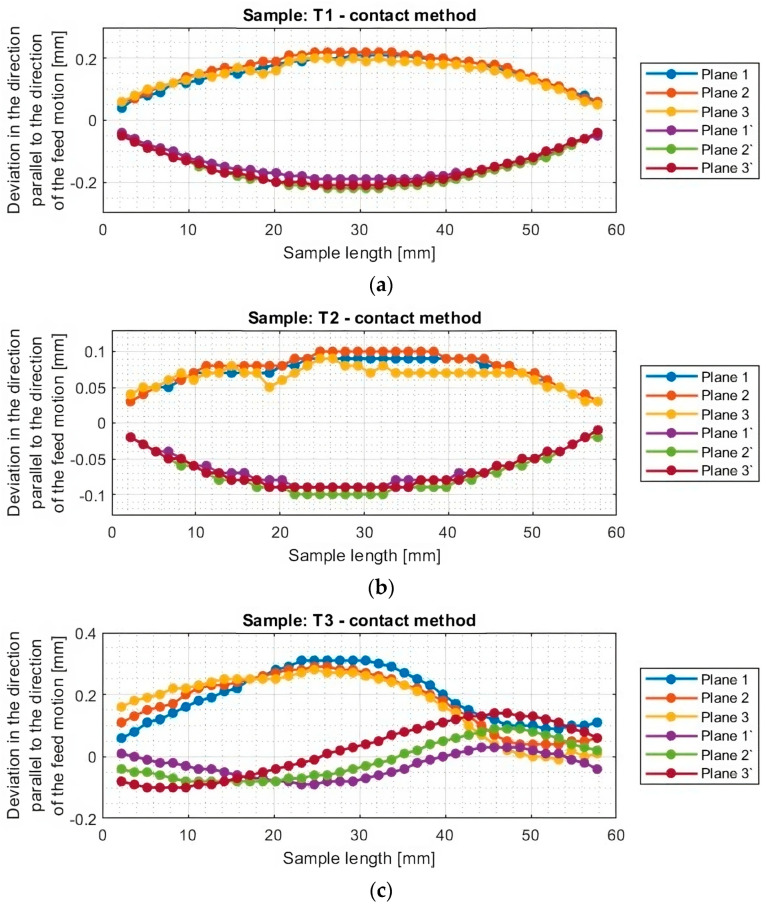
Deviations of the horizontal thin wall in the direction parallel to the direction of tool feed motion for titanium alloy samples machined using adaptive face milling strategy, determined by contact method: (**a**) T1; (**b**) T2; (**c**) T3.

**Figure 15:**
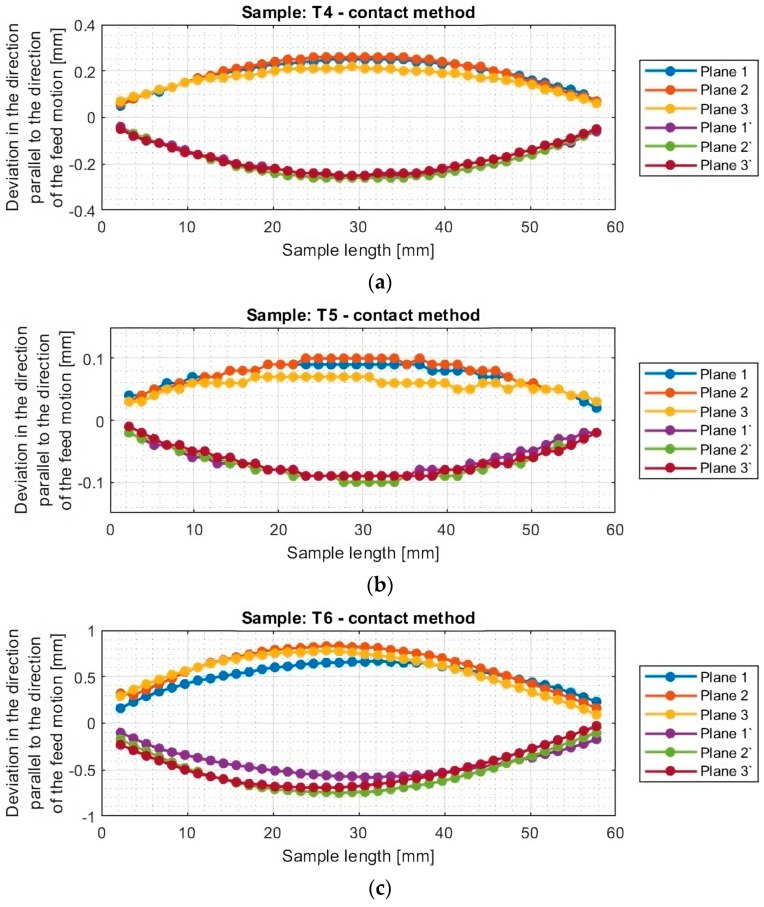
Deviations of the horizontal thin wall in the direction parallel to the direction of tool feed motion for titanium alloy samples machined using adaptive cylindrical milling strategy, determined by contact method: (**a**) T4; (**b**) T5; (**c**) T6.

**Figure 16:**
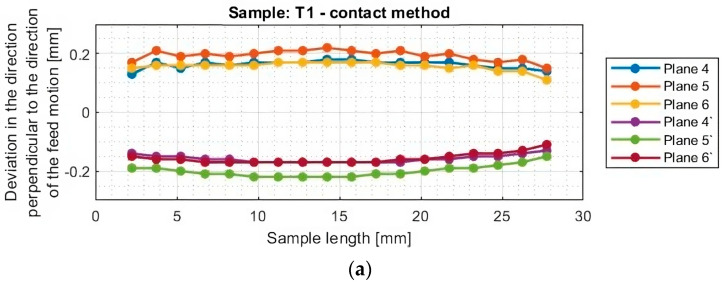

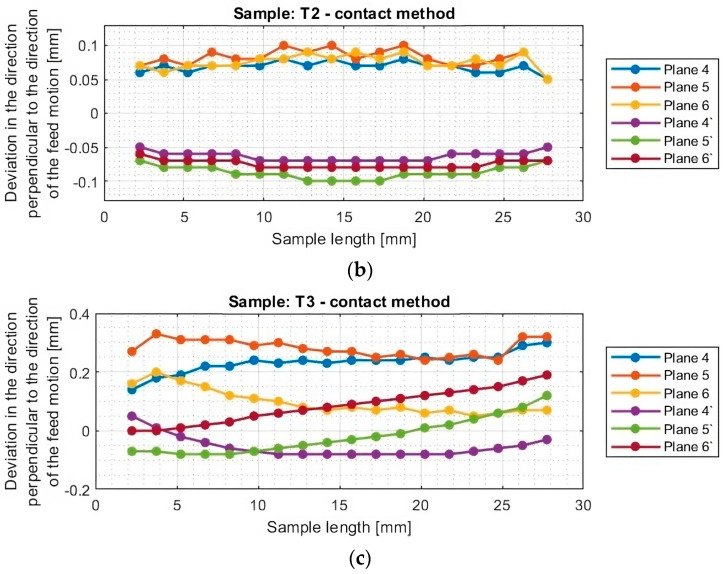
Deviations of the horizontal thin wall in the direction perpendicular to the direction of tool feed motion for titanium alloy samples machined using adaptive face milling strategy, determined by contact method: (**a**) T1; (**b**) T2; (**c**) T3.

**Figure 17:**
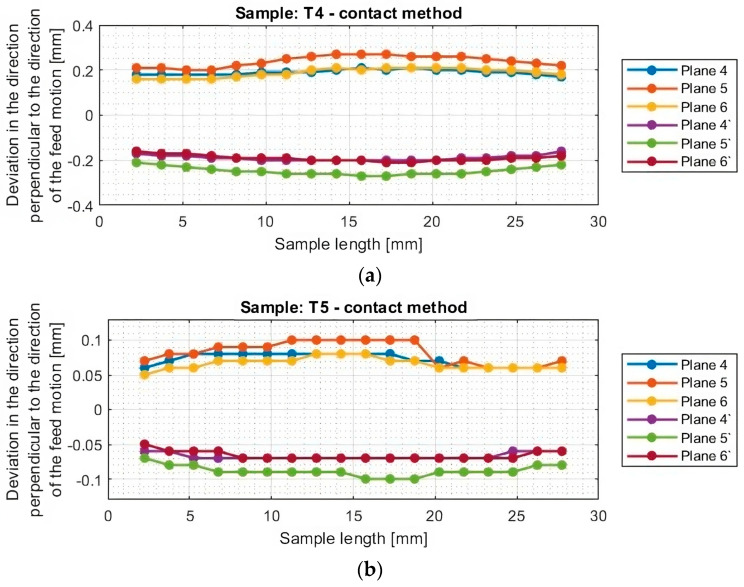

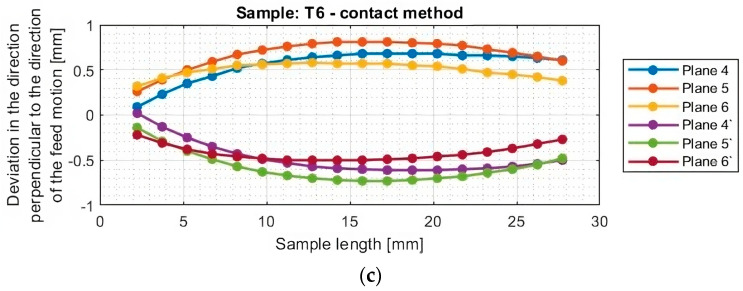
Deviations of the horizontal thin wall in the direction perpendicular to the direction of tool feed motion for titanium alloy samples machined using adaptive cylindrical milling strategy, determined by contact method: (**a**) T4; (**b**) T5; (**c**) T6.

In the second part, the measurement of dimensional and shape accuracy was carried out using a coordinate measuring machine following the conditions described in Section 2.4. The measurement results for individual samples, obtained using the contact method, were obtained in a manner analogous to the results obtained using the optical method. Diagrams of deviations in the direction parallel to the direction of the tool feed motion for titanium alloy samples determined by the contact method are shown in Figure 14 and Figure 15, while diagrams of deviations in the perpendicular direction are shown in Figure 16 and Figure 17.

### 3.3. Correlation of the Obtained Results

A study of dimensional and shape accuracy by two methods—optical and contact—was carried out for Ti6Al4V titanium alloy samples with thin walls in the horizontal orientation. The analysis of the deviations of the thin walls, based on the presented graphs, revealed their similarity in nature and shape between the discussed measurement methods. In many cases, even a convergence of results was observed. On this basis, it is concluded that the measurements carried out and the mathematical models used in the development of the measurement program and report are correct. Despite the convergence, slight differences in the results between the two methods may have their origin in the accuracy of the measuring machines or possible differences in the way of sample basing. Such observations, despite their presence, do not seem to significantly affect the overall correctness and reliability of the results obtained. However, for absolute certainty, the differences in deviation values between the described methods (optical and contact) will be determined.

**Figure 18:**
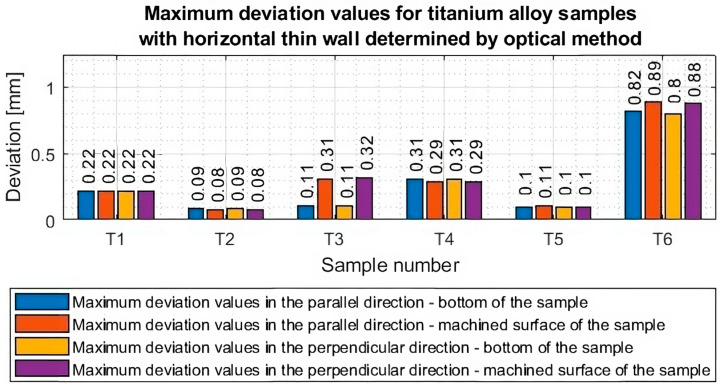
Values of maximum deviations from the bottom side of the sample and the machined surface in both directions of measurement, determined by the optical method.

**Figure 19:**
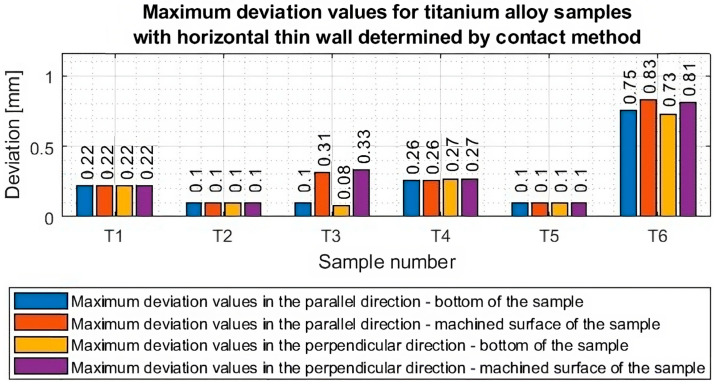
Values of maximum deviations from the bottom side of the sample and the machined surface in both directions of measurement, determined by the contact method.

Based on the plots of the deviations of the thin wall in a horizontal orientation in the selected planes, shown in Figure 10, Figure 11, Figure 12, Figure 13, Figure 14, Figure 15, Figure 16 and Figure 17, plots of the maximum deviations of the bottom of the sample and the machined surface in both directions determined using the optical method (Figure 18) and the contact method (Figure 19) have been prepared. 

Based on the presented graphs of maximum deviations of individual samples (Figure 18 and Figure 19), the occurring measurement differences between the optical and contact methods are shown in Table 5.

Based on the data in Table 4, it can be seen that a maximum error of about 8% was obtained. The largest discrepancy occurred for sample T6, for which the thin-walled surface was deformed and damaged after machining, so the apparent deviation may be the result of slightly different point coordinates during the determination of the bases. It should be borne in mind that the maximum measurement discrepancy we determined is the difference between the largest deviation values. The overall shape of the graphs in many cases is comparable, indicating relatively similar results. It is worth noting that the discrepancy increased as recorded deviations increased. 

External factors during the transportation of the sample and mounting may also have affected the accuracy of the measurement. The thin-walled specimens presented in the study are characterized by reduced stiffness, so the dimensions may have changed slightly between measurements. An additional factor affecting the discrepancies obtained is the presence of measurement uncertainty, as well as the deviation accuracy used during the measurements. In further studies, it would be advisable to adopt a higher accuracy (e.g., to the third decimal place), which would allow a more detailed determination of the differences in the obtained results.

The study [45] compared different measurement methods (coordinate measuring machine, 3D optical scanner, and 3D computed tomography) when studying the dimensional deviations of an aluminum cube that did not contain thin-walled structures. Comparison of linear dimensions after CMM and optical scanner measurements allowed a maximum difference of 0.05 mm, which, according to the authors, gives convergent results. The differences that we determined for thin-walled samples are similar to those presented in the cited study, which allows us to conclude that the results converge and the 3D optical scanner can be effectively used to measure small parts containing thin-walled structures.

As a rule of thumb, the use of contact CMMs leads to measurement results with higher precision, especially in the context of current technological advances. The choice of measurement method should be justified by the geometric tolerances adopted for the product. The use of contact methods in the context of CMMs makes it possible to achieve greater measurement precision; however, in some cases, it is limited to inspection at specific points, with extended measurement time. Within the framework of the analyzed issue presented in this paper, it is worth noting that the optical method using a 3D optical scanner is extremely sensitive to various conditions, such as surface contamination or the size of the analyzed object. Nevertheless, its advantage is that it can accurately scan the entire surface of the sample and analyze any point or area even after the measurement is completed. It is also worth noting that the measurement process itself using the optical scanner is much shorter compared to currently available contact methods.

## 4. Summary and Conclusions

Within the framework of the present study, samples from titanium alloy Ti6Al4V containing a thin wall in horizontal orientation were prepared. For the samples obtained in this way, the dimensional and shape accuracy was measured by determining the deviations of the thin wall in selected cross-sections. The measurement was carried out using an optical method with a 3D optical scanner and a contact method using a coordinate measuring machine. The correlation was carried out to evaluate the applicability of the 3D optical scanner (GOM) for measuring thin-wall structures of small dimensions.

Based on the presented deviation graphs, the following conclusions can be given:

- Lower deviation values were obtained for samples machined using adaptive face milling (T1–T3) compared to adaptive cylindrical milling (T4–T6).
- The lowest value of maximum deviation (equal to 0.09) was obtained for the T2 sample machined with the tool for high-performance machining using adaptive face milling. For this sample, smaller deviation values were observed compared with the obtained values in the cited publications [20,21,22,23,24]. However, it should be borne in mind that the cited papers presented thin-wall deviations in a vertical orientation—it was noted that there was a leading lack of deviation results for titanium alloy samples with a thin wall in a horizontal orientation.
- The highest value of the maximum deviation (equal to 0.89) was obtained for the T6 sample made with the tool for high-speed machining using adaptive cylindrical milling. The deviation value for this one is significantly higher than the results obtained for the other samples—nine times higher than the sample with the lowest deviations (T2) and three times higher than the values obtained for the sample made with the same tool but with the opposite strategy, i.e., adaptive face milling (T3).
- For samples T1–T2 and T4–T6, a thin-wall thickness close to the assumed thickness of 1 mm was obtained.
- For sample T3 (tool for high-speed machining, adaptive face milling), the graphs are shifted concerning the horizontal y = 0 axis by about 0.2 mm towards positive values, so the resulting wall thickness is smaller by this value than assumed. The thin wall for this sample was very deformed (twisted) after machining.
- High stability was observed in the course of deviation values for samples milled with the tool for general purposes (samples: T1 and T4) and the tool for high-performance machining (samples: T2 and T5).

A cross-correlation of the results showed a qualitative similarity in the distribution of dimensional deviations of samples with horizontal thin walls measured using the optical method and the contact method. A maximum measurement discrepancy of about 8% was obtained between the used measurement methods. Based on this, the consistency of the results obtained when measuring with a 3D optical scanner was confirmed. This is a valuable observation, as it confirms the thesis that proper selection of measurement conditions allows the study of thin-walled structures of small dimensions with relatively good accuracy.

Based on ongoing experiments, we see the following potential research directions:

- Expanding the experiment to include samples containing other shapes of thin-walled structures.
- Use of other variants of cutting parameters.
- Conducting tests on samples containing different thin-wall thicknesses.
- Use of other methods to support the material during machining.

## Figures and Tables

**Table 1:** Cutting parameters adopted during the experiment.

Sample	Material	Tool	Machining Strategy	Depth of Cut a_p_ (mm)	Radial Depth a_e_ (mm)
T1	Titanium alloy—Ti6Al4V	JS554100E2R050.0Z4-SIRA	adaptive face milling	2 (3 passes)	4
T2		JS754100E2C.0Z4A-HXT		2 (3 passes)	4
T3		JH730100D2R100.0Z7-HXT		2 (3 passes)	4
T4		JS554100E2R050.0Z4-SIRA	adaptive cylindrical milling	6 (1 pass)	1.33
T5		JS754100E2C.0Z4A-HXT		6 (1 pass)	1.33
T6		JH730100D2R100.0Z7-HXT		6 (1 pass)	1.33

**Table 4:** Comparison of 3D optical scanner and coordinate measuring machine.

Methods/Machine	Benefits and Advantages	Limitations and Disadvantages
Optical method—3D optical scanner	Much faster measurement compared to, for example, CMM, which reduces costs; Ability to measure surfaces with complex shapes; Ability to check individual dimensions after the measurement program is completed due to the possession of a point cloud and, as a result, deviations between actual coordinates and model values; Ability to change basing during measurement evaluation; Ability to map the actual surface as a point cloud.	The surface to be measured must be clean and free of dirt; It is required to stick reference points on the object to be measured and to cover the object with a special anti-reflection preparation; Method limited to reflective materials; It is necessary to use a measuring baseplate; Difficult to measure holes, pockets, and thin parts due to the inability to project enough striations; It is necessary to make a suitable foundation for the machine since the method is sensitive to vibrations.
Contact method—coordinate measuring machine	High accuracy of measurement; Ability to measure any material; In many cases, no need for additional instrumentation (when measuring in the free state); Ability to easily measure thin-walled structures.	Measurement only at selected measurement sites (measurement in a point or linear manner); No possibility of inspecting the product after the measurement is completed; No ability to change measurement strategy and bases after measurement.

**Table 5:** Measurement differences of maximum deviation values between optical and contact methods for titanium alloy samples.

Sample	T1	T2	T3	T4	T5	T6
Difference (mm)	0	0.02	0.03	0.05	0.01	0.07

## Data Availability

Data are contained within the article.

## Appendix A

An example of a color map for sample T1 with a horizontal thin wall obtained during measurement using the 3D optical scanner is shown in Figure A1.
